# Effect of replacing concentrates with cassava root-top silage on feed utilization, rumen fermentation, blood parameters and growth performance in beef cattle

**DOI:** 10.5713/ab.24.0076

**Published:** 2024-08-16

**Authors:** Nirawan Gunun, Randorn Phimda, Nonthasak Piamphon, Walailuck Kaewwongsa, Darunee Puangbut, Chatchai Kaewpila, Waroon Khota, Anusorn Cherdthong, Pongsatorn Gunun

**Affiliations:** 1 Department of Animal Science, Faculty of Technology, Udon Thani Rajabhat University, Udon Thani 41000, Thailand; 2 Department of Animal Science, Faculty of Natural Resources, Rajamangala University of Technology Isan, Sakon Nakhon Campus, Phangkhon, Sakon Nakhon 47160, Thailand; 3 Department of Animal Science, Faculty of Agriculture and Natural Resources, Rajamangala University of Technology Tawan-ok, Bangpra, Sriracha, Chonburi 20110, Thailand; 4 Agriculture Program, Faculty of Technology, Udon Thani Rajabhat University, Udon Thani 41000, Thailand; 5 Tropical Feed Resources Research and Development Center (TROFREC), Department of Animal Science, Faculty of Agriculture, Khon Kaen University, Khon Kaen 40002, Thailand

**Keywords:** Beef Cattle, Blood Parameters, Cassava Root-top Silage, Feed Utilization, Growth Performance, Rumen Fermentation

## Abstract

**Objective:**

This experiment aimed to evaluate the effects of replacing concentrates with cassava root-top silage (CARTOS) on feed intake, digestibility, rumen fermentation, blood parameters, and growth performance of beef cattle.

**Methods:**

Twenty crossbred bulls with a body weight (BW) of 226±56 kg were randomly assigned to one of five treatments for 90 d in a randomized complete block design having four blocks based on BW. The concentrates were replaced by CARTOS at levels of 0%, 25%, 50%, 75%, and 100% dry matter (DM). Animals were fed dietary treatments at 1.8% BW, with rice straw offered *ad libitum*.

**Results:**

The DM and crude protein (CP) intake were decreased (p<0.01, p = 0.04) when the diet’s CARTOS level was increased. The digestibility of DM, OM, and CP were not different among treatments, while fiber digestibility was increased with the inclusion of CARTOS (p = 0.03). The addition of CARTOS to replace concentrates did not change ruminal pH or volatile fatty acid proportions except for acetic acid, which increased with the addition of CARTOS (p = 0.03). The ruminal ammonia-nitrogen (NH_3_-N) was decreased (p<0.01) with increasing levels of CARTOS. The blood glucose and blood urea nitrogen decreased (p = 0.01) with the addition of CARTOS at 100%, whereas total protein and hematological parameters did not change with increasing levels of CARTOS. The use of CARTOS to substitute concentrates at 75% and 100% decreased average daily gain (ADG) and gain to feed ratio (G:F) (p<0.01); therefore, the addition of CARTOS up to 50% maintained ADG and G:F in beef cattle.

**Conclusion:**

CARTOS can replace concentrates up to 50% in beef cattle diets without adversely affecting feed intake, nutrient digestibility, rumen fermentation characteristics, blood parameters, or growth performance of beef cattle.

## INTRODUCTION

Smallholder farmers have largely supported the production of beef cattle in Southeast Asia, especially Thailand. The most important feed resources for ruminants are grasses and legumes. However, ruminants are fed low-quality roughage, particularly rice straw, during the dry season. Feeding high concentrates enhances feed utilization, rumen fermentation characteristics, and growth performance in ruminants [1]. However, the increased price of feedstuffs, particularly soybean meal and cassava chip, has led to an increase in concentrate prices and a decrease in farmers’ profits. There is a substantial amount of interest in the research and development of alternative feedstuffs to substitute for concentrates.

Cassava ( *Manihot esculenta*) is mostly used in Africa and tropical Asia for starch production, staple foods, and livestock feeds. Cassava chips are rich in starch and are considered the primary energy source for ruminants. However, the demand for cassava chip fed to animals is not sufficient during the rainy season, and their price has been increasing. Moreover, the low price of cassava root and an unorganized marketing system have contributed to the unprofitability of cassava farming. Cassava tops are a by-product of cassava cultivation, and they consist of leaves, petioles, and stems at 61.6%, 20.0%, and 18.4% dry matter (DM), respectively [2]. Cassava tops contain 23.0% crude protein (CP) [3] and they have the potential to be used as a protein source in ruminant feed. The utilization of cassava products as feed has been limited due to their high content of hydrogen cyanide (HCN), which could result in negative effects on ruminants [4]. Fresh cassava root and cassava top contain 99 to 189 and 100 to 333 mg/kg DM, respectively [5–7]. Sun-drying can lower the HCN content of cassava. However, collecting cassava leaves during the rainy season makes sun-drying difficult; therefore, ensiling is considered the best preservation method.

The cassava root is a good energy source, and the cassava top can be useful as a protein source in ruminant feed. If it can be developed into an incorporated cassava root-top, it may replace concentrates and reduce the cost of feed. Amos et al [8] suggested that an ensiled cassava root-top (CARTOS) ratio of 70:30 had a 10.1% CP and 4,180 kcal/kg gross energy and had the potential to replace corn in the swine diet. Bureenok et al [9] reported that the cassava root was ensiled with cassava top at a ratio of 60:40, using 20% DM in the total mixed ration, resulting in increased feed intake and milk yield in dairy cows. Gunun et al (unpublished) using an *in vitro* system, reported that the use of a cassava root to cassava top ratio of 40:60 is suitable for use as animal feed. The study hypothesized that CARTOS could serve as an alternate feed source to substitute concentrates without adversely affecting feed utilization, rumen fermentation, blood parameters, or the growth performance of beef cattle. Therefore, the purpose of this study was to determine the effect of replacing concentrates with CARTOS on feed intake, digestibility, rumen fermentation, blood chemistry, hematology, and growth performance in beef cattle.

## MATERIALS AND METHODS

### Animal care

The Animals Ethical Committee of Rajamangala University of Technology Isan approved all of the experimental animals and methodology used in this study (approval number 04-66-001). Permission and consent were obtained from the owner of the beef cattle farm of the livestock community enterprise group to conduct the animal experiment.

### Preparation of CARTOS

Fresh cassava root and cassava top were harvested at 10 mos from smallholders’ cassava production in Sangsawang, Nong Saeng, Udon Thani, Thailand. The top of the cassava was collected for a length of 50 cm, including the young stems, leaves, and petioles, and was then chopped to 2 to 3 cm by a chopping machine (Samyodmotor Co., Ltd., Ubon Ratchathani, Thailand). Fresh cassava root was also chopped into chips. Using a cassava root-to-cassava top ratio of 40:60 as suggested by our earlier research (Gunun et al, unpublished) the CARTOS mixture (without any additive) was then stored in plastic drums (150 L capacity, 95 cm height, 45 cm diameter) with a capacity of about 120 kg, then closed with a plastic lid and lever lock ring. All silos were kept indoors at ambient temperature (23°C to 38°C) for at least 14 d before feeding. The ensiling of CARTOS was carried out every 14 d in all experimental periods. After 14 d of ensiling, all silos were opened to measure the pH of the silage with a portable pH meter (FiveGo, Mettler-Toledo GmbH, Greifensee, Switzerland), and CARTOS samples were taken to determine the fermentation end product and chemical composition.

### Animals, treatments, and experimental design

This research was carried out at a beef cattle farm of the livestock community enterprise group, Sangsawang, Nong Saeng, Udon Thani, Thailand. Twenty crossbred (Brahman×Thai native) bulls with a body weight (BW) of 226±56 kg were divided into four blocks according to BW. Within each block, the cattle were randomly assigned to one of five dietary treatments. A randomized complete block design (RCBD) was used in this study to replace concentrates (Table 1) with CARTOS at 0%, 25%, 50%, 75%, and 100% on a DM basis. The concentrates were formulated according to the NRC recommendations [10]. Animals were utilized to assess performance during a 90 d period. The cattle were fed dietary treatments at a rate of 1.8% of their BW, along with rice straw *ad libitum*, in two equal feeding times at 07:00 h and 16:00 h. The cattle were always kept in separate pens (3×4 m) with concrete floors and a wooden fence. The animals were provided with clean water and mineral blocks. The mineral blocks contained NaCl 995.11 g/kg, Na 390.00 g/kg, Mg 2.00 g/kg, Zn 0.81 g/kg, Cu 0.22 g/kg, I 0.10 g/kg, and Se 0.01 g/kg (KNZ, Arnhem, Netherlands).

### Data collection and sampling procedures

Cattle were weighed at the initial BW and the final BW at 90 d to calculate the average daily gain (ADG). Feed offered and refusals each morning were noted and collected for chemical analysis. During the last five days (d 86 to 90 of the trial), feces were taken from each animal to test digestibility. Rectal sampling was used to collect samples of fresh feces (about 500 g) in the 06:00 h and 15:00 h. Composite samples of each animal’s daily fresh feces were mixed and chilled at 4°C.

The samples, which included CARTOS, concentrates, rice straw, CARTOS to concentrate ratios, refusals, and feces, were dried at 60°C in a hot air oven and ground (1-millimeter screen using Cyclotech Mill; Tecator, Hoganas, Sweden). The concentrations of ash, CP [11], neutral detergent fiber (NDF), and acid detergent fiber (ADF) [12] were analyzed. A modified vanillin-HCl method [13] was used to measure the amount of condensed tannins (CT) in CARTOS. The amounts of organic acids (lactic acid, acetic acid, propionic acid, and butyric acid) in CARTOS were assessed using a periodic acid reagent [14] and gas chromatography (Nexis GC-2030, Shizuku Co., Kyoto, Japan). Acid-insoluble ash was used as an internal marker to assess the nutrient digestibility [15]. The digestibility coefficients of those nutrients were calculated as 100–100×(% indicator in feed/% indicator in feces)×(% nutrient in feces/% nutrient in feed) [15].

On the final day of the experiment, at 4 h post-feeding, 200 mL of rumen fluid was collected with a stomach tube connected to a vacuum pump. To avoid contamination with saliva, the first 100 mL of ruminal samples were discarded. The samples were then filtered through four layers of cheesecloth and immediately measured using a portable pH meter (FiveGo, Mettler-Toledo GmbH, Greifensee, Switzerland). Samples of ruminal fluid were centrifuged at a speed of 16,000×g for 15 min at 4°C, and the supernatant was refrigerated at −20°C. The ruminal samples were thawed before being tested for ammonia-nitrogen (NH_3_-N) (Kjeltech Auto 1030 Analyzer; Tecator, Sweden) [16] and volatile fatty acid (VFA) using gas chromatography (Nexis GC-2030; Shimadzu Co., Kyoto, Japan) [17].

Blood samples of about 10 mL from the jugular vein were collected from each cattle at the same time as ruminal fluid samples. Glucose, total protein, and blood urea nitrogen (BUN) were measured with a chemical analyzer (Mindray BS-600; Shenzhen Mindray Bio-medical Electronics Co., Ltd., Shenzhen, China). The blood’s hemoglobin, hematocrit, white blood cells (WBC), neutrophils, lymphocytes, monocytes, and eosinophils were measured with a hematology analyzer (Mindray BC-3000 Plus; Shenzhen Mindray Bio-medical Electronics Co., Ltd., China).

### Statistical analysis

The data were analyzed by the MIXED procedure of SAS [18]. Data about feed intake and growth performance were analyzed with repeated measurements according to the following model:


$$Y_{ijkl}=\mu +\alpha_{i}+\beta_{j}+\gamma_{jk}+\delta_{l}+\alpha \delta_{il}+{ɛ}_{ijkl},$$

where *Y_ijkl_* is the dependent variable, *μ* is the overall mean, *α_i_* is the fixed effect of the *i*-th treatment (Trt) (*i* = 1 to 5), *β_j_* is the random effect of *j*-th block of initial BW (*j* = 1 to 4), *γ_jk_* is the random effect of *k*-th cattle nested within the *j*-th block of initial BW (*k* = 1 to 20), *δ_l_* is the fixed effect of the *l*-th time (month) of the experiment (*l* = 1 to 3), *αδ_il_* is the fixed effect of the *i*-th treatment by the *l*-th time of the experiment interaction, and *ɛ_ijkl_* is the residual error. The most appropriate covariance structure for repeated measures was selected based on the least Bayesian information criterion (BIC) value. Initial BW was used as a covariate. The least-squares means were separated using the PDIFF option. Treatment trends were statistically compared using orthogonal polynomial contrasts (linear, quadratic, and cubic). Statistical significance was accepted at p<0.05.

## RESULTS

### Chemical composition of diets

The replacement of concentrates with CARTOS slightly reduces the concentrations of DM and CP (Table 2). The NDF and ADF were increased with increasing levels of CARTOS. Moreover, higher levels of CARTOS as a substitute concentrate tend to decrease feed costs.

### Feed intake and growth performance

Roughage intake was decreased in CARTOS to replace concentrates at 25% to 100% (Trt, p = 0.03; time, p<0.01) (Table 3). The increasing CARTOS at 75% and 100% lower concentrate intake (Trt, p = 0.03; time, p<0.01) with interaction of treatment and time effect (p<0.01). Hence, the addition of CARTOS at 25% to 100% decreased the total intake of beef cattle (Trt, p<0.01; time, p<0.01). Initial and final BW did not differ among treatments. The increasing levels of CARTOS at 75% and 100% decreased ADG (Trt, p<0.01; time, p = 0.03), resulting in an interaction effect (Trt×time, p = 0.02) (Table 3). In addition, the G:F was lower in CARTOS to replace concentrates at 75% and 100% (Trt, p<0.01; time, p<0.01).

### Nutrient intake and digestibility

The intake of OM, NDF, and ADF did not differ between treatments. The increasing CARTOS to replace concentrates at 100% lowers the intake of CP (p = 0.04) (Table 4). The NDF and ADF digestibility increased (p = 0.03) with the addition of CARTOS at 100% in the diet.

### Rumen fermentation

The ruminal pH and total VFA were not different among treatments (Table 5). The NH_3_-N levels decreased with the addition of CARTOS at 25% to 100% (p<0.01). Acetic acid (C2) was increased quadratically (p = 0.01), and the inclusion of CARTOS at 25% to 75% showed higher acetic acid than the other treatment. Nevertheless, no differences were observed in terms of propionic acid (C3), butyric acid (C4), or C2:C3.

### Blood chemicals and hematological parameters

The concentrations of total protein, hemoglobin, hematocrit, WBC, neutrophils, lymphocytes, monocytes, and eosinophils were similar among dietary treatments (Table 6). The glucose and BUN were lower with the inclusion of CARTOS at 100% (p = 0.02).

## DISCUSSION

Ensiled cassava root-top ratio of 40:60 has a CP content of 13.6% (Table 1). This finding is consistent with previous research, which found that the CP of cassava root-top silage at a 50:50 ratio was 13.0% [8]. The pH of CARTOS was 4.7, consistent with Kang et al [19] finding that the cassava top silage pH ranges from 3.7 to 4.8. Additionally, previous studies showed that cassava root silage pH was 4.0 [20]. During ensiling, microbial protease degradation of proteins converts them into non-protein nitrogen (NPN) compounds (peptides, amino acids, and NH_3_-N), leading to a higher silage pH in forage [21]. These results could be due to the high protein content in CARTOS, which might lead to increased degradation of protein to NPN, which increases silage pH. The CARTOS contained NDF and ADF at 43.6% and 33.8% DM, respectively. The results indicated a slight increase in NDF and ADF content with the inclusion of CARTOS to replace the concentrates.

It is commonly known that the higher NDF concentration (>35.5%) in feed decreased the voluntary feed intake of ruminants [22]. In the current study, CARTOS replaced concentrates, which decreased DM intake. The high fiber content of cassava tops is because they consist of leaves, petioles, and young stems. Combining the cassava top and root resulted in a mixture with higher levels of NDF and ADF than the concentrates, which decreased DM intake. In addition, adding CARTOS to replace concentrates could lower CP in diets, thereby reducing CP intake in beef cattle. Previous studies found that the inclusion of cassava top silage in the diet enhanced fiber digestibility in beef cattle [1]. These are consistent with our study, which found that the digestibility of NDF and ADF was enhanced with the replacement of concentrates by CARTOS at 75% and 100%. The fiber digestibility was improved due to the slower ruminal rate of passage, and longer rumen retention time was associated with higher fiber content in the diets. In addition, plants contain CT, which may enhance the digestibility of protein in animal feeds. Protein-tannin complexes are insoluble in the rumen; nevertheless, they dissociate in the abomasum, allowing hydrolyzed protein and amino acids to be absorbed in the small intestine. Adding CARTOS to replace concentrate did not affect the digestibility of CP. Similarly, Suharti et al [23] reported that sheep fed bitter cassava leaf meal at 15% and 30% to substitute concentrate did not change CP digestibility in sheep. According to Viennasay et al [3], the inclusion of cassava top silage as a roughage source improved the digestibility of CP, nitrogen (N) intake, N retention, and urinary N in dairy steers. Koenig and Beauchemin [24] reported that the addition of CT extract to diets containing corn distillers’ grains increased fecal N intake and reduced N in urine. These findings may be due to cattle’s increasing N output into their feces and urine, which did not affect CP total tract digestibility with the inclusion of CARTOS.

This study found that ruminal pH did not differ among treatments. The optimal pH range for rumen microorganisms was 6.5 to 7.0 [25], and the rumen pH range for all treatments was 6.8 to 7.0. Ruminal NH_3_-N concentrations for dietary treatments range from 16.3 to 28.0 mg/dL, which is closer to the optimum level (15 to 30 mg/dL) of NH_3_-N for microbial growth in the rumen [26]. Moreover, ruminal NH_3_-N concentrations were reduced with increasing levels of CARTOS. The CT and proteins combine to form complexes in the rumen. These complexes decrease ruminal protein degradation and NH_3_-N concentrations. The CARTOS contained CT at 5.4%. These could be due to CT in cassava top and protein binding, which reduce ruminal NH_3_-N concentrations in our study. Another explanation is that adding CARTOS to replace concentrates produced a lower CP content in the diets and lower CP intake, resulting in reduced ruminal NH_3_-N concentrations. The levels of NH_3_-N had a relationship with BUN concentrations. The ruminal NH_3_-N is absorbed into portal vein blood and converted to urea-nitrogen in the liver. In the current study, cattle consuming CARTOS diets had a lower level of BUN.

The VFA serve as energy sources that contribute to the ruminant’s production performance; therefore, it was essential to investigate their metabolism. Acetic acid is one of the main products of the fermentation of structural carbohydrates by rumen microbes. The inclusion of CARTOS resulted in a quadratic increase in the molar proportion of acetic acid. Moreover, the addition of CARTOS at 25% to 75% increased acetic acid. It is plausible that the cellulolytic bacterial degradation and fermentation of structural carbohydrates in CARTOS produce acetic acid in the rumen.

Hematological analysis is frequently used to determine an animal’s health and nutrition. The addition of CARTOS did not affect the hematology status. In addition, previous research indicates that hematological values in cattle blood are within the range of acceptable levels [27]. Glucose is an important nutrient for all animal organisms. When including higher structural carbohydrate from CARTOS to replace concentrates at 0%, 25%, 50%, 75%, and 100%, the concentration of blood glucose is 85.2, 82.2, 80.2, 79.0, and 70.2 mg/dL, respectively. The inclusion of CARTOS at 100% results in the lowest blood glucose concentration. The CARTOS are high in fiber content and lower in starch polysaccharides. This might reduce the glucose concentration in the blood of cattle fed CARTOS to replace concentrates at 100%. Lower glucose concentrations are correlated with hypoinsulinemia, an insulin deficiency that stimulates the release of fatty acids from adipose tissue, leading to an increase in the production of ketone bodies [28]. The decreased levels of glucose and increasing ketone bodies may be affected by reduced feed intake, as shown in our study.

Beef cattle production efficiency depends on both ADG and feed efficiency. In the current study, the ADG and G:F were lower in beef cattle fed CARTOS to replacement concentrates at 75% and 100%, respectively. In agreement with the findings of our study, previous research found that higher fiber content in diets reduced performance in beef cattle [27]. Glucose in the blood is related to growth performance and carcass quality in animals. These results could be related to the high fiber content of feed, which decreased nutrient intake and blood glucose when CARTOS continually replaced concentrates, leading to a decrease in beef cattle growth performance and feed utilization. In addition, the hydrolysis of cyanogenic glycosides releases large amounts of cyanide, which is poisonous to ruminants. Hydrogen cyanide, which is toxic when ingested by animals in amounts greater than 200 mg/kg of fresh matter, is the reason for the cassava product’s limited use [4]. Ruminants tend to be more susceptible to being poisoned by cyanide than those of monogastric species. A previous study found that replacing concentrates with bitter cassava leaf meal at 15% and 30% increased HCN intake, which reduced the rumen fermentation, especially total VFA and propionic acid, leading to lower ADG and feed efficiency in sheep [23]. The decrease in cyanide levels during the ensiling process may be caused by the release of glycosides and free cyanide, leading to the production of a substantial amount of gas effluent. Khota et al [21] found that the cassava root silage reduced the total cyanide to 36% of the initial value. In previous reports, the concentration of HCN at ensiling cassava root and casava leaf silage was reported at 88.0 [29] and 72.0 mg/kg [3], respectively. Although adding the highest levels of CARTOS did not change the amount of total VFA or propionic acid in the rumen, cattle may receive the higher concentration of HCN from CARTOS, resulting in decreased growth performance. However, it is not clear because our study didn’t determine HCN concentration in CARTOS, HCN intake, blood thiocyanate, etc. in cattle.

## CONCLUSION

The replacement of concentrates with CARTOS did not alter hematological parameters. The inclusion of CARTOS resulted in a lower feed intake, growth performance, ruminal NH_3_-N concentration, blood glucose, and BUN concentration. The addition of CARTOS increases fiber digestibility and acetic acid production. Adding up to 50% CARTOS to replace concentrates without negative effects on feed utilization, rumen fermentation characteristics, blood metabolites, hematological status, and growth performance in beef cattle.

## Figures and Tables

**Table 1:** Chemical composition of concentrates, cassava root-top silage (CARTOS) and rice straw

Items	Concentrates	Rice straw	CARTOS
Ingredient (% DM)			
Cassava chip	51.0		
Soybean meal	17.0		
Rice bran	15.0		
Dried brewers’ grains	14.0		
Molasses	2.0		
Salt	0.5		
Mineral and vitamin mixture	0.5		
Chemical composition			
Dry matter (% as fed)	90.4±0.28	93.6±0.18	34.8±0.46
Organic matter (% DM)	91.2±0.60	91.5±0.09	93.0±0.60
Crude protein (% DM)	14.5±0.35	3.1±0.14	13.6±0.27
Neutral detergent fiber (% DM)	31.9±0.62	70.4±0.22	43.6±0.45
Acid detergent fiber (% DM)	19.6±0.58	55.5±0.51	33.8±0.52
Condensed tannins (% DM)	-	-	5.4±0.03
pH	-	-	4.7±0.06
Lactic acid (g/kg DM)	-	-	83.0±14.5
Acetic acid (g/kg DM)	-	-	37.9±3.17
Propionic acid (g/kg DM)	-	-	0.16±0.03
Butyric acid (g/kg DM)	-	-	0.07±0.05

**Table 2:** Chemical composition and feed costs of the experiment diets

Items	CARTOS replacing concentrates (%)				

	0	25	50	75	100
Chemical composition					
DM (% as fed)	90.4±0.28	67.4±0.30	62.8±0.50	55.6±0.62	34.8±0.46
Organic matter (% DM)	91.2±0.60	91.9±0.11	91.5±0.11	92.2±0.09	93.0±0.28
Crude protein (% DM)	14.5±0.35	14.3±0.33	14.1±0.22	13.8±0.17	13.6±0.27
Neutral detergent fiber (% DM)	31.9±0.62	37.0±0.56	39.3±0.60	45.2±0.52	43.6±0.45
Acid detergent fiber (% DM)	19.6±0.58	22.5±0.60	22.7±0.34	32.4±0.40	33.8±0.52
Feed costs (USD/100 kg DM)	32.3	26.9	21.4	16.0	10.6

CARTOS, cassava root-top silage; DM, dry matter.

**Table 3:** Effect of cassava root-top silage (CARTOS) replacing concentrates on feed intake and growth performance in beef cattle

Items	CARTOS replacing concentrates (%)					SEM	p-value^1)^			Contrast		
		
	0	25	50	75	100		Trt	Time	Trt×time	L	Q	C
Dry matter intake (kg/d)												
Roughage	2.0	1.6	1.5	1.6	1.5	0.09	0.03	<0.01	0.55	0.01	0.07	0.14
Concentrates	4.7	4.4	4.7	4.3	4.0	0.12	0.03	<0.01	<0.01	<0.01	0.32	0.22
Total intake	6.7	5.9	6.2	5.9	5.5	0.14	<0.01	<0.01	0.20	<0.01	0.57	0.02
Growth performance												
ADG (kg/d)	1.1	0.8	0.9	0.7	0.5	0.08	<0.01	0.03	0.02	<0.01	0.98	0.25
G:F	0.17	0.14	0.14	0.12	0.09	0.01	<0.01	<0.01	0.10	<0.01	0.65	0.51
BW (kg)												
Initial	226.5	236.5	230.0	219.5	219.0	21.15	0.97	-	-	0.63	0.75	0.69
Final	328.0	313.0	310.5	281.5	266.0	25.43	0.44	-	-	0.77	0.99	0.71

SEM, standard error of the mean; L, linear; Q, quadratic; C, cubic; ADG, average daily gain; G:F, gain to feed ratio; BW, body weight.

1) T = treatment; Time = 1, 2, and 3 month of experiment.

**Table 4:** Effect of cassava root-top silage (CARTOS) replacing concentrates on nutrient intake and digestibility in growing beef cattle

Items	CARTOS replacing concentrates (%)					SEM	Trt	Contrast		
	
	0	25	50	75	100			L	Q	C
Nutrient intake (kg/d)										
Organic matter	6.4	5.8	6.0	5.3	4.9	0.56	0.37	0.07	0.75	0.72
Crude protein	0.8	0.7	0.8	0.7	0.6	0.06	0.04	0.02	0.55	0.45
Neutral detergent fiber	2.9	2.9	3.1	2.9	2.7	0.31	0.93	0.58	0.57	0.74
Acid detergent fiber	2.0	1.9	2.0	2.2	2.0	0.23	0.95	0.71	0.97	0.50
Digestibility coefficients (%)										
Dry matter	59.9	59.6	58.0	59.1	63.0	2.09	0.54	0.40	0.17	0.55
Organic matter	63.6	63.5	61.7	63.2	66.5	1.99	0.56	0.39	0.19	0.60
Crude protein	51.1	55.4	50.2	48.0	57.7	4.58	0.58	0.69	0.43	0.16
Neutral detergent fiber	48.1	48.9	50.7	50.2	59.3	2.84	0.03	0.02	0.20	0.55
Acid detergent fiber	42.2	36.9	39.1	42.8	53.5	3.60	0.03	0.01	0.03	0.96

SEM, standard error of the mean; Trt, treatment; L, linear; Q, quadratic; C, cubic.

**Table 5:** Effect of cassava root-top silage (CARTOS) replacing concentrates on rumen fermentation in beef cattle

Items	CARTOS replacing concentrates (%)					SEM	Trt	Contrast		
	
	0	25	50	75	100			L	Q	C
pH	6.8	6.9	7.0	6.9	7.1	0.09	0.29	0.10	0.90	0.33
NH_3_-N (mg/dL)	28.0	21.0	19.8	16.3	14.0	1.31	<0.01	<0.01	0.17	0.27
Total VFA (mmol/L)	62.9	54.8	52.9	58.0	53.6	3.42	0.27	0.17	0.28	0.16
VFA (mol/100 mol)										
Acetic acid (C2)	62.2	65.1	63.9	64.9	62.0	0.79	0.03	0.81	0.01	0.93
Propionic acid (C3)	20.7	19.3	21.2	19.5	22.5	1.37	0.46	0.38	0.32	0.74
Butyric acid (C4)	17.1	15.6	14.9	15.6	15.5	1.14	0.72	0.38	0.35	0.64
C2:C3	3.1	3.4	3.0	3.4	2.8	0.20	0.25	0.30	0.19	0.67

SEM, standard error of the mean; Trt, treatment; L, linear; Q, quadratic; C, cubic; VFA, volatile fatty acid.

**Table 6:** Effect of cassava root-top silage (CARTOS) replacing concentrates on blood chemistry and hematology in beef cattle

Items	CARTOS replacing concentrates (%)					SEM	Trt	Contrast		
	
	0	25	50	75	100			L	Q	C
Glucose (mg/dL)	85.3	82.3	80.3	79.0	70.3	2.93	0.02	<0.01	0.34	0.37
Total protein (g/dL)	6.2	6.3	6.8	6.2	5.8	0.30	0.30	0.33	0.09	0.81
BUN (mg/dL)	9.5	9.0	7.0	8.8	3.3	1.32	0.02	<0.01	0.22	0.18
Hemoglobin (g/dL)	6.1	8.2	6.9	8.0	6.7	0.70	0.20	0.67	0.09	0.62
Hematocrit (%)	18.3	24.8	20.8	24.0	20.0	2.11	0.21	0.68	0.10	0.63
White blood cells (10^9^/L)	17.9	18.4	18.5	17.1	13.6	2.24	0.54	0.19	0.29	0.82
Neutrophils (%)	53.5	61.5	52.8	42.9	60.8	5.30	0.08	0.37	0.45	0.05
Lymphocytes (%)	46.5	38.5	44.3	54.9	39.3	5.56	0.10	0.45	0.30	0.07
Monocytes (%)	0.0	0.0	1.3	1.3	0.0	0.39	0.06	0.33	0.05	0.46
Eosinophils (%)	0.0	0.0	1.3	0.5	0.0	0.57	0.48	0.78	0.18	0.58

SEM, standard error of the mean; Trt, treatment; L, linear; Q, quadratic; C, cubic; BUN, blood urea nitrogen.
